# Supplementary material for: Patterns of genomic evolution in advanced melanoma
Source: Nat Commun. 2018 Jul 10;9:2665. doi: 10.1038/s41467-018-05063-1 (PMC6039447; doi:10.1038/s41467-018-05063-1)
Supplement: Supplementary file 1 — Supplementary Information [file 41467_2018_5063_MOESM1_ESM.docx]

# Supplementary Information

# Patterns of genomic evolution in advanced melanoma

Birkeland et al.

Supplementary Figures 1-15

## Supplementary Figures


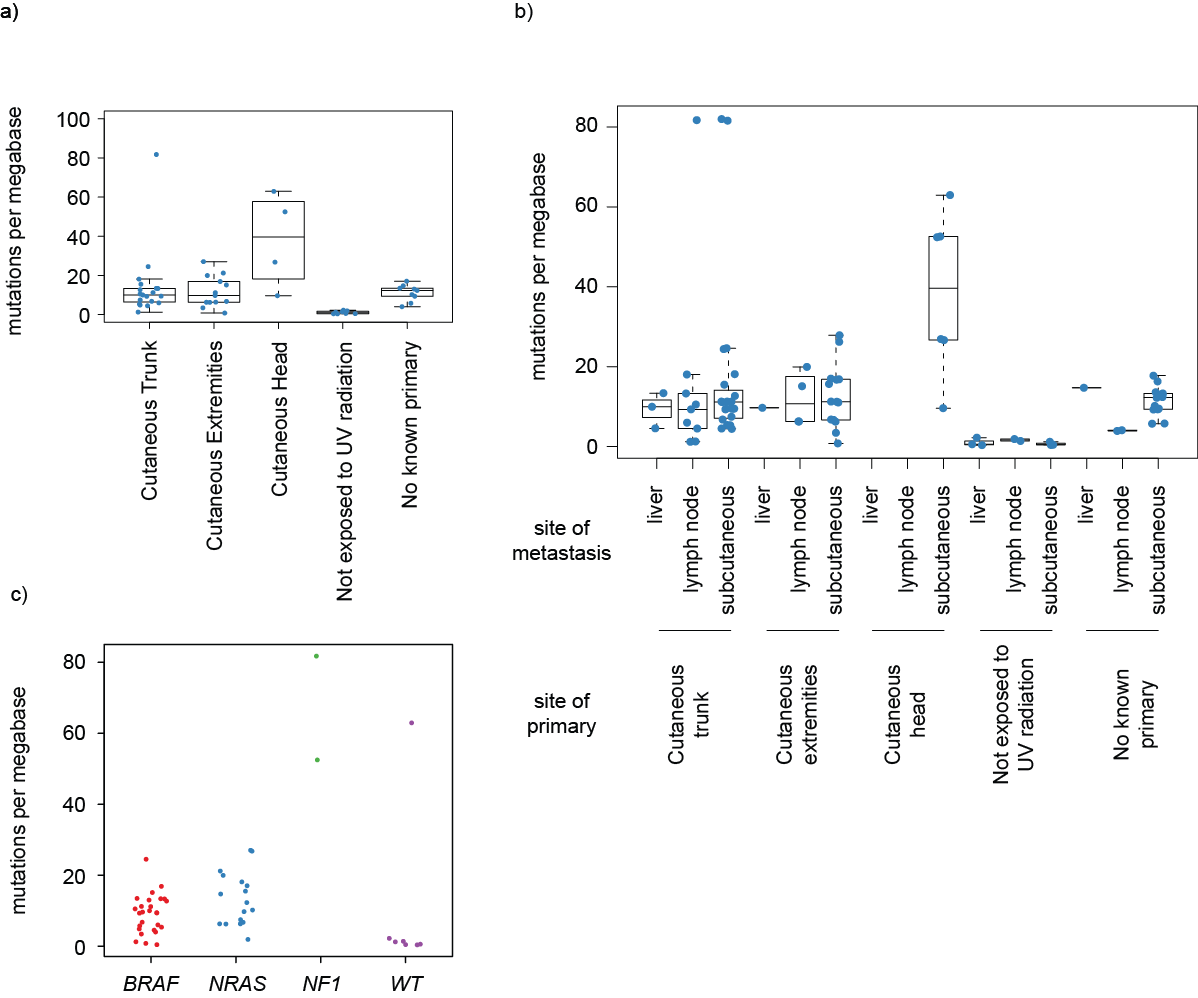


**Supplementary Figure 1**: Unique mutation per patient: **a**) number of mutations per patient (average of samples) in coding regions per megabase according to site of the primary lesion. The category “Not exposed” includes mucosal (n=2), acral (n=3), uveal (n=2), and one patient with a skin lesion that was situated perianally. **b**) The number of mutations per lesion according to the site of the lesion the patient’s primary tumor. Boxes with whiskers span the interquartile range (IQR), with whiskers extending to 1.5 times the IQR from the upper and lower bounds of the boxes. **c**) The number of unique mutations per patient according to driver mutation status of *BRAF*, *NRAS* and *NF1*.


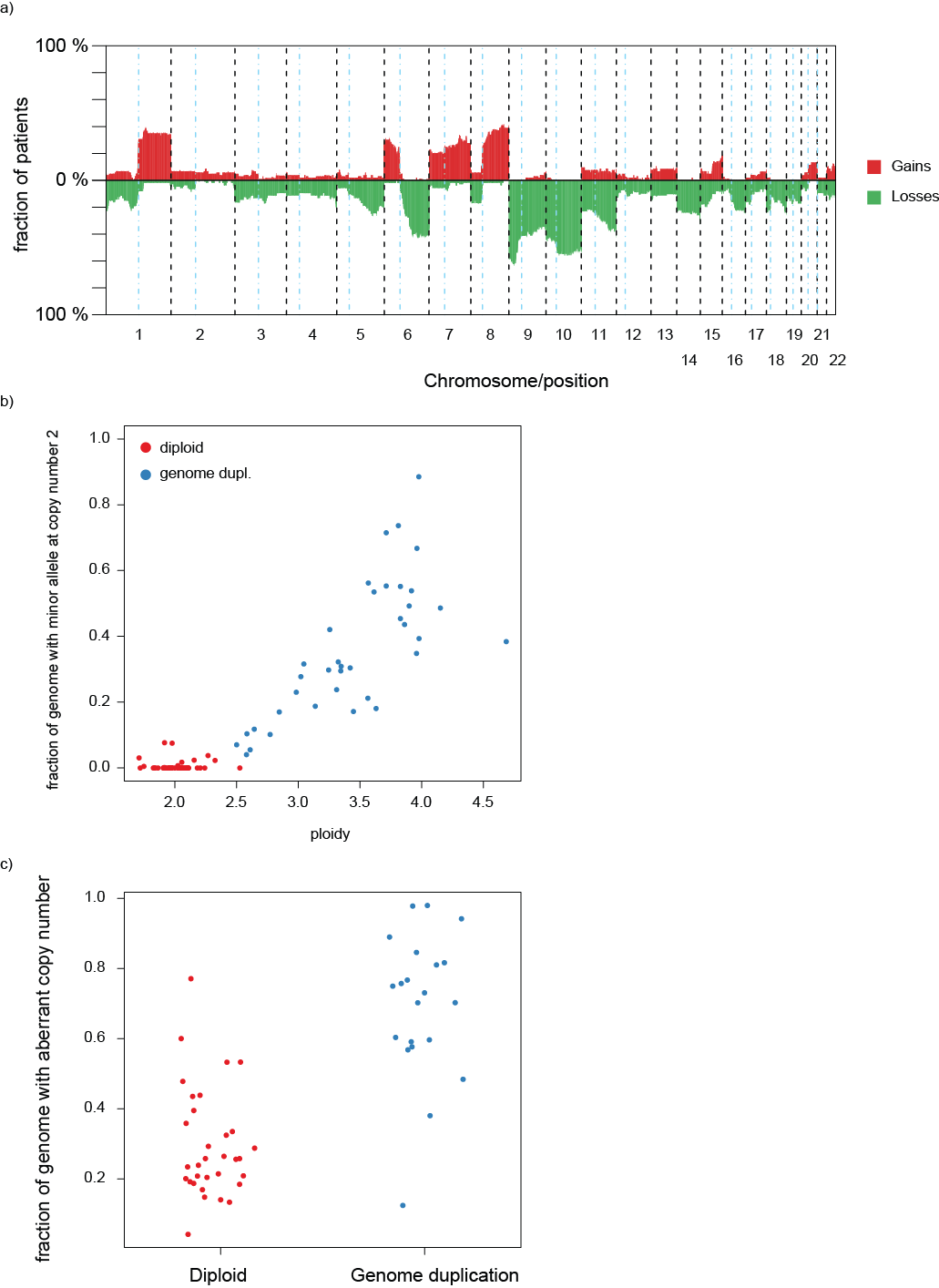


**Supplementary Figure 2**: Genomic complexity and genome duplication. **a**) Prevalence of copy number gains and losses across the genome. Gain or loss for any region was defined as ≥3 copies in total, or ≤1 copy in total, respectively. For patients with genome duplication, the respective thresholds were ≥6 and ≤2. For patients with more than one analyzed sample, the fraction of samples with gain or loss was recorded. **b**) Using the fraction of the genome where the minor allele was at copy number 2 in combination with ploidy, we categorized patients as having undergone a genome duplication event (blue) or not (red). **c**) Measuring genomic complexity as the fraction of the genome not at a balanced copy number of 2 (diploid) or 4 (genome duplicated), we compared patients with diploid tumors to patients with tumors having undergone genome duplication. For patients with multiple samples, we used the average value of the patient’s samples.


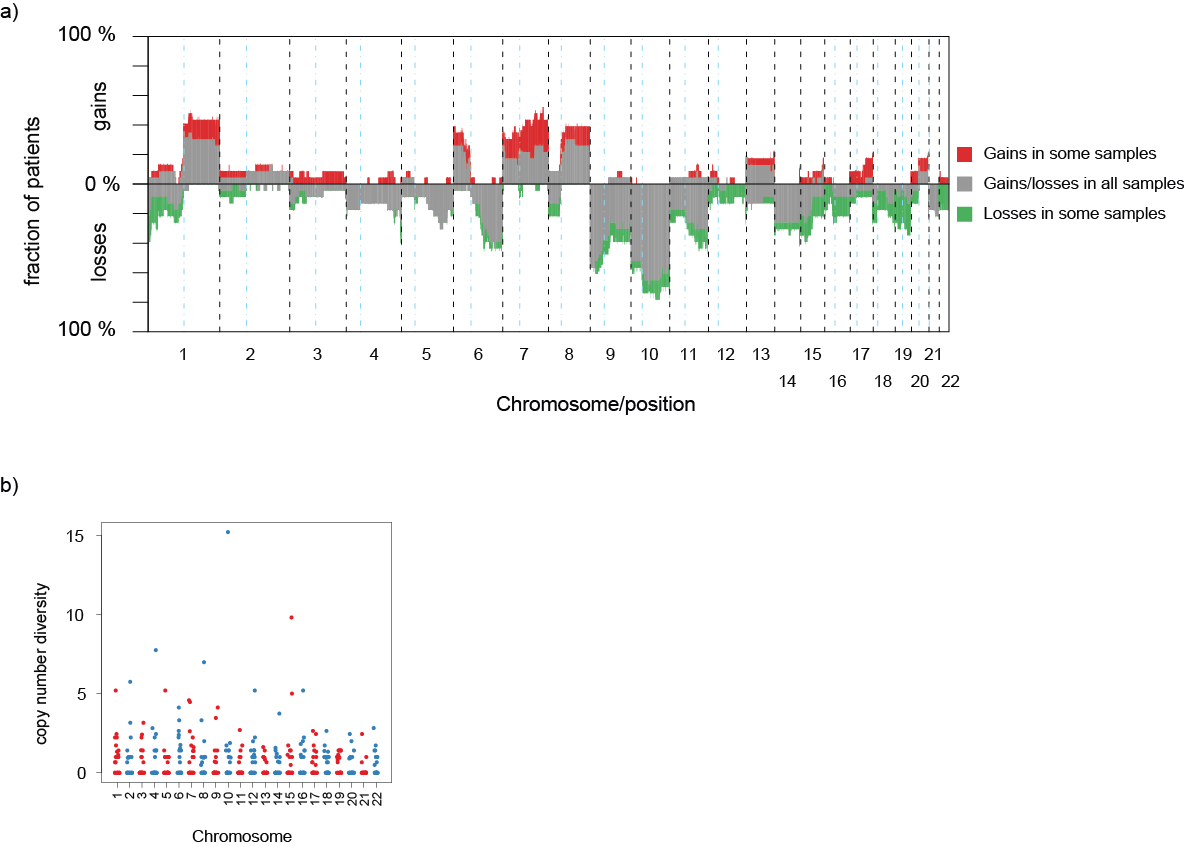


**Supplementary Figure 3**: Copy number diversity. **a**) Prevalence of heterogeneous copy number gains and losses across the genome. Gains and losses are defined as in figure S2. Grey fields represent the fraction of patients with gains or losses in all individual samples. Red and green fields represent the fraction of patients with heterogeneous copy number gains and losses, respectively. Only patients with multiple sampled lesions are included in this figure. **b**) The copy number diversity according to chromosome; each patient is represented by a point per chromosome.


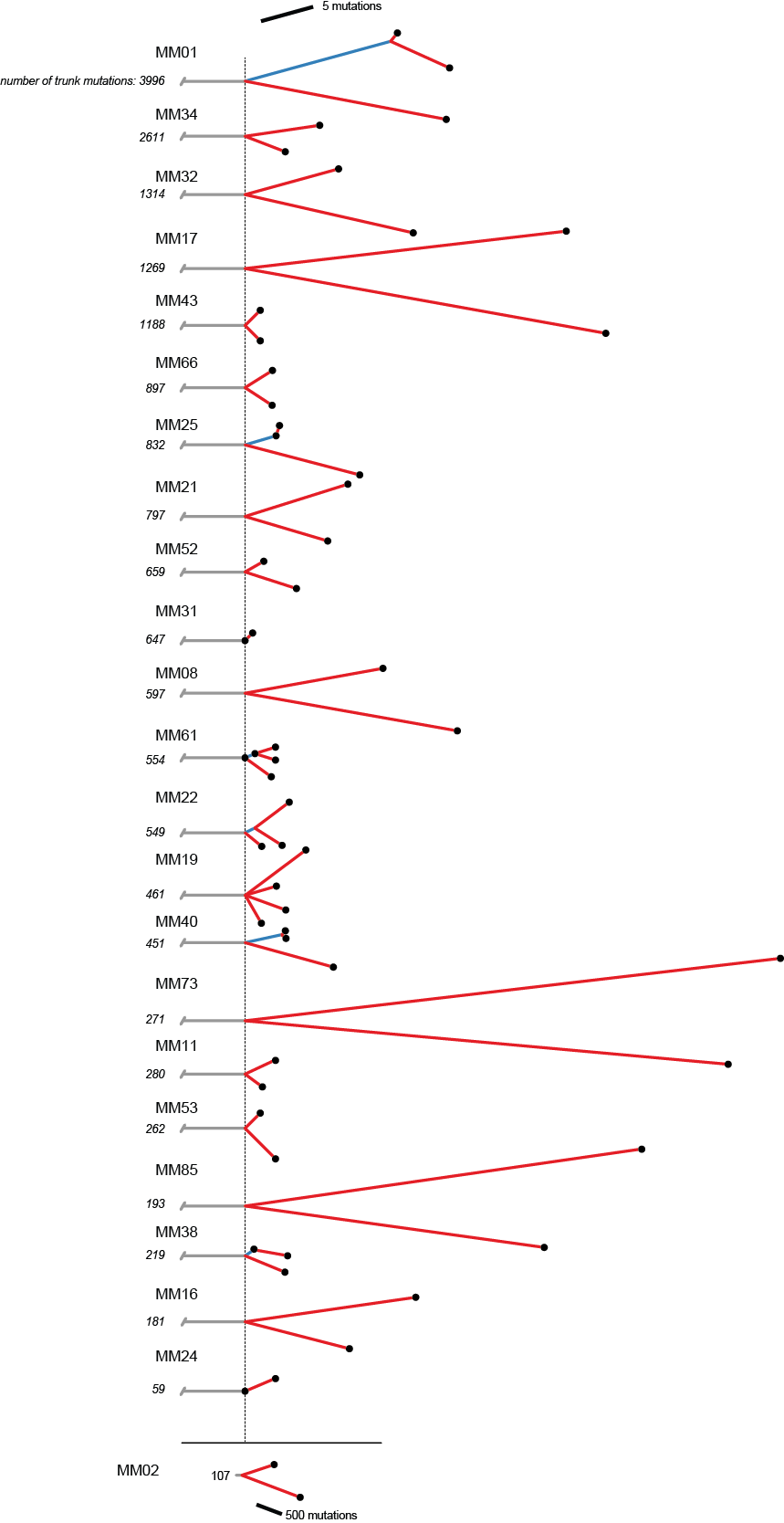


**Supplementary Figure 4 (previous page)**: Phylogenetic trees showing the relationships between samples collected from the same patients. The phylogenetic trees are derived from the presence or absence of mutations across samples as depicted in figure 2b and c in the main text. Sampled lesions are indicated by black dots, and the color of branches match the color code in figure 2b and c: grey = trunk; blue = branch; red = private mutations. The trunks of the phylogenetic trees have been truncated, with the total number of mutations indicated next to the base of each tree. Branch lengths are proportional to the numbers of mutations specific to each branch, with the number of mutations indicated by a scale bar. Patient MM02 is depicted separately due to the high number of branch mutations in the samples from this patient.


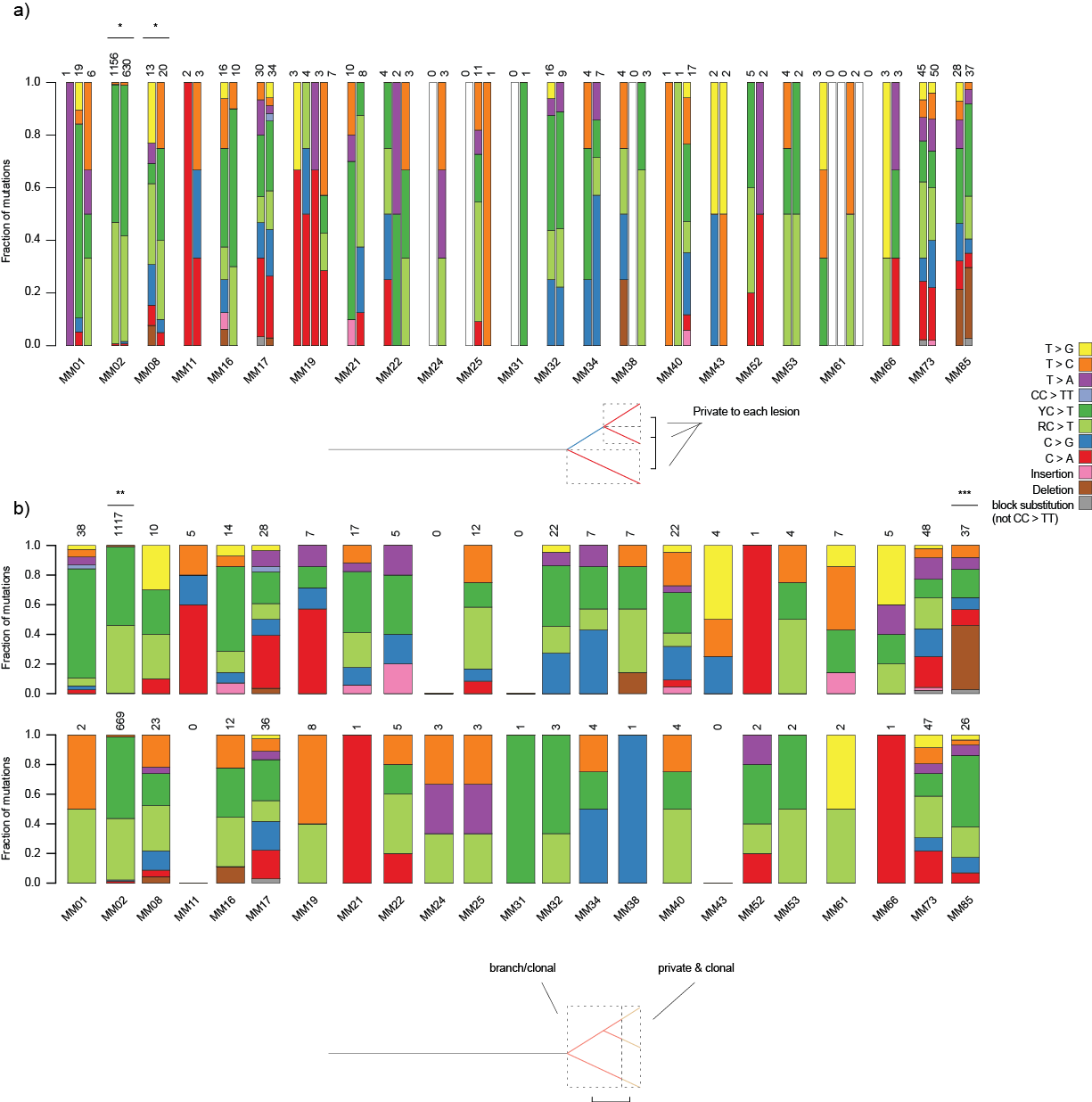


**Supplementary Figure 5**: Mutation type distribution of branch mutations for each sample. **a**) Private mutations per lesion in each patient, or (**b**) branch mutations per patient according to status as subclonal are compared as portrayed in diagrams below barplots. Blank columns represent samples without mutations in the relevant category. The numbers of mutations are shown above each column. Asterisks indicate significant differences according to fisher exact test (or chi-square tests in the case of high numbers of mutations).


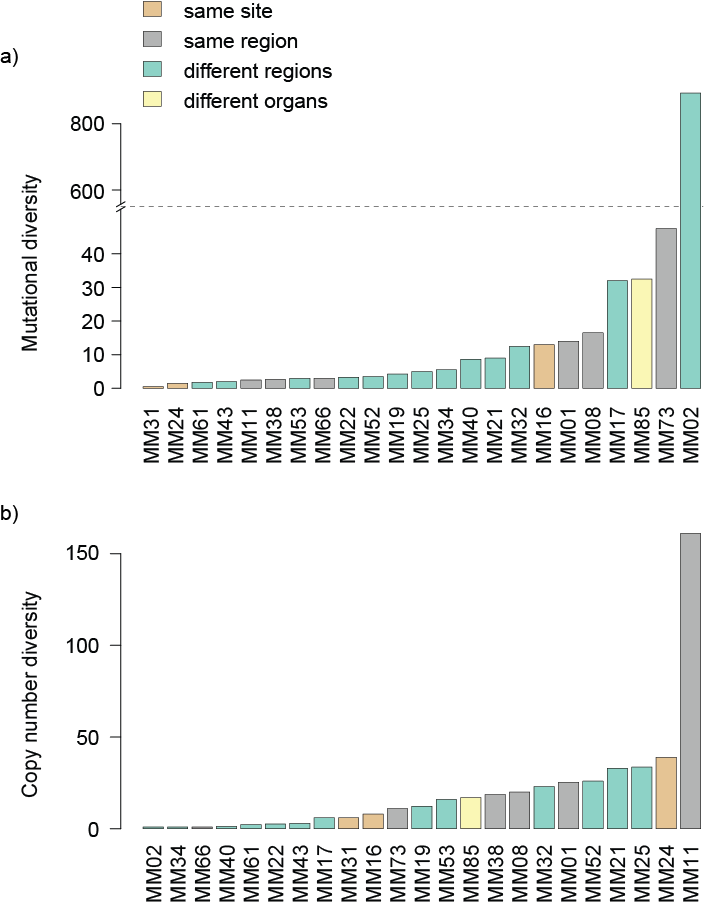


**Supplementary Figure 6**: mutational (**a**) and copy number (**b**) diversity according to the anatomical diversity of sampled lesions. Patients were categorized according to the anatomical distance between biopsy sites. For patients with more than two samples, the largest distance was used. Anatomical distance was categorized as same site, with samples taken from the same lesion at different time points; same region, defined as lesions in areas draining to the same lymph nodes; different regions; or different organs. Subcutaneous and lymph node deposits were not considered as separate organs by this classification.


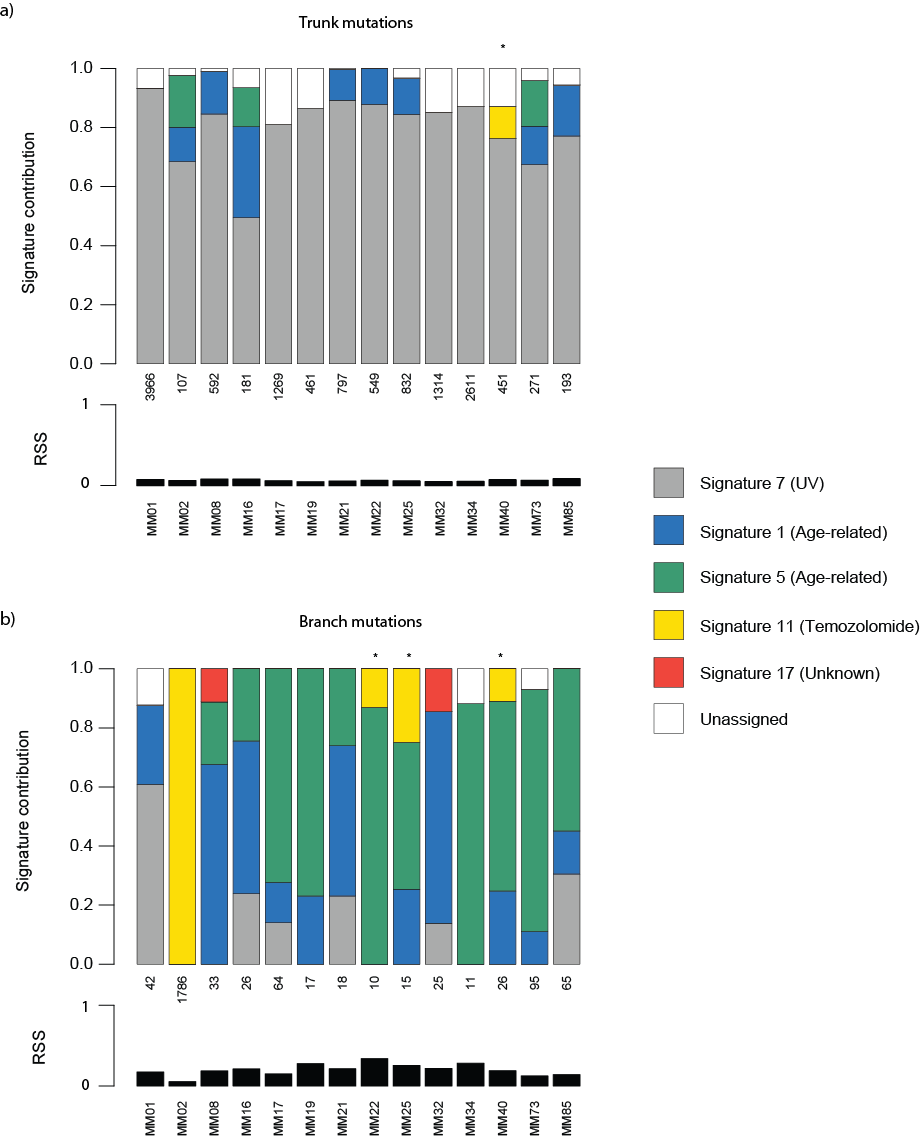


**Supplementary Figure 7**: Estimated contributions of mutational processes to (**a**) trunk and (**b**) branch mutations for each patient in which the total number of branch mutations was over 10 (the number of trunk or branch mutations is shown under each bar). The residual sum of squares (RSS) is shown below, which is a measure of how closely the observed mutations match the estimated process contributions. Only contributions of 5 mutational processes were assessed, and mutational signatures corresponding to less than 10% of mutations were not considered; thus, for some patients, the mutational signature contributions do not sum to 1.0. *Although some mutations in these patients were predicted to be caused by alkylating agent exposure, only two of these samples had been exposed to such therapy, each predicted to have less than five mutations assigned to this source. We therefore consider this attribution to be by chance.


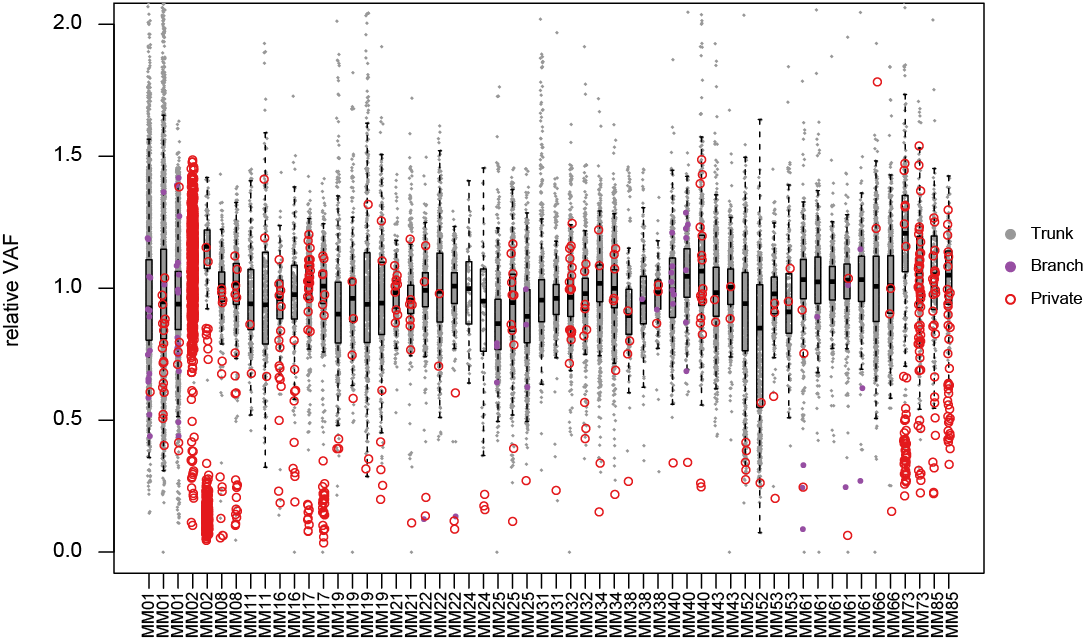


**Supplementary Figure 8**: Relative variant allele frequency (VAF) of mutations according to heterogeneity and subclonality status. Relative VAF represents the variant allele frequency corrected by tumor purity and local copy number, and is thus analogous to cellular prevalence. Mutations are color-coded according to status as trunk (found in all lesions; gray), branch (found in some, but not all lesions; purple), and private (found uniquely in one lesion; red). Boxes span the interquartile range and whiskers extend to 1.5 times the interquartile range from the bounds of the boxes.


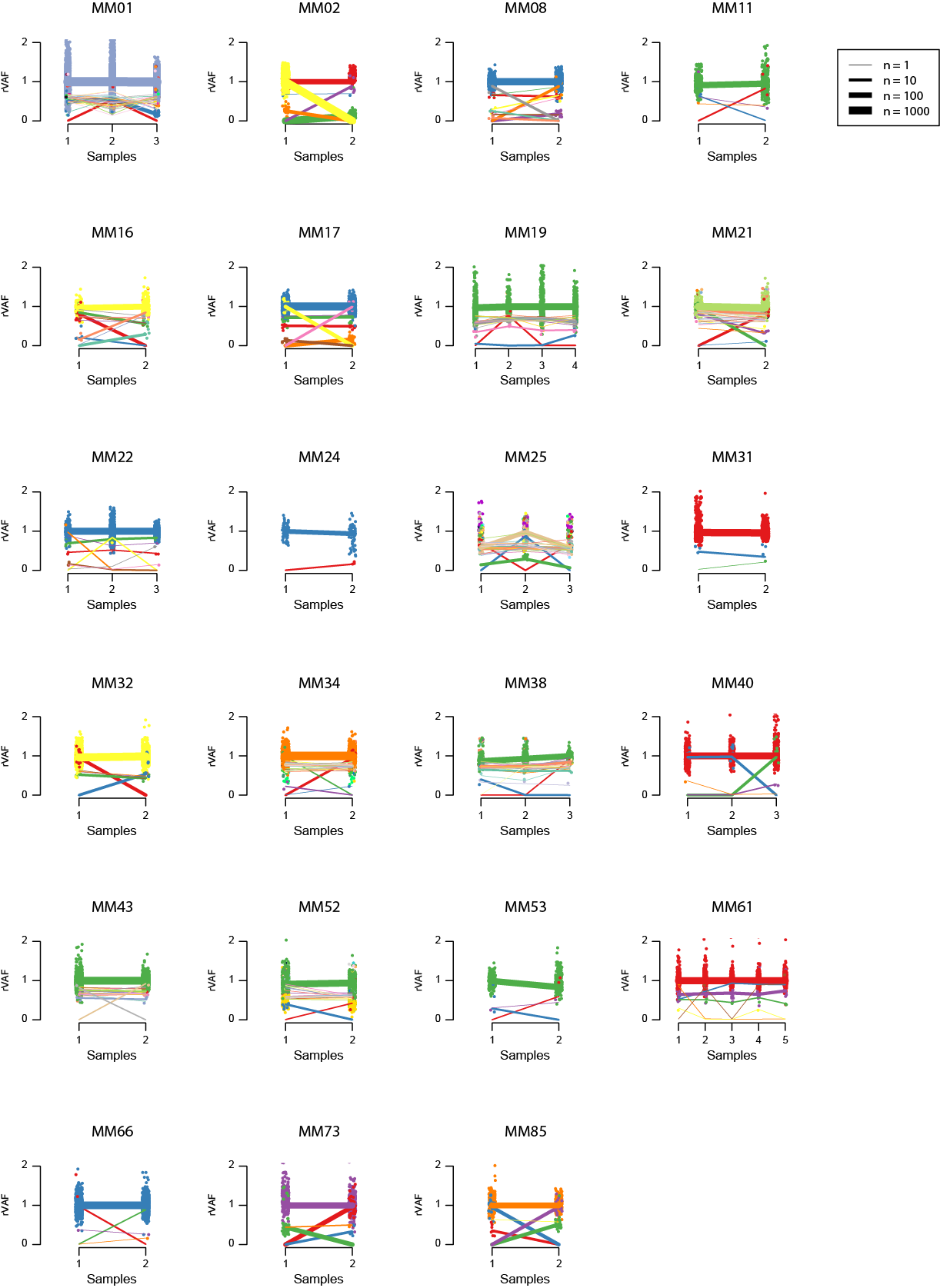


**Supplementary Figure 9**: Cross sample clustering of mutations according to cellular prevalence using PyClone. Mutations and their relative variant allele frequencies (rVAF) are indicated for each sample with dots, colored according to the cellular population to which they were predicted to belong. The predicted cellular prevalence of each population of cells is indicated by lines, the weight of which correspond to the number of mutations belonging to each population.


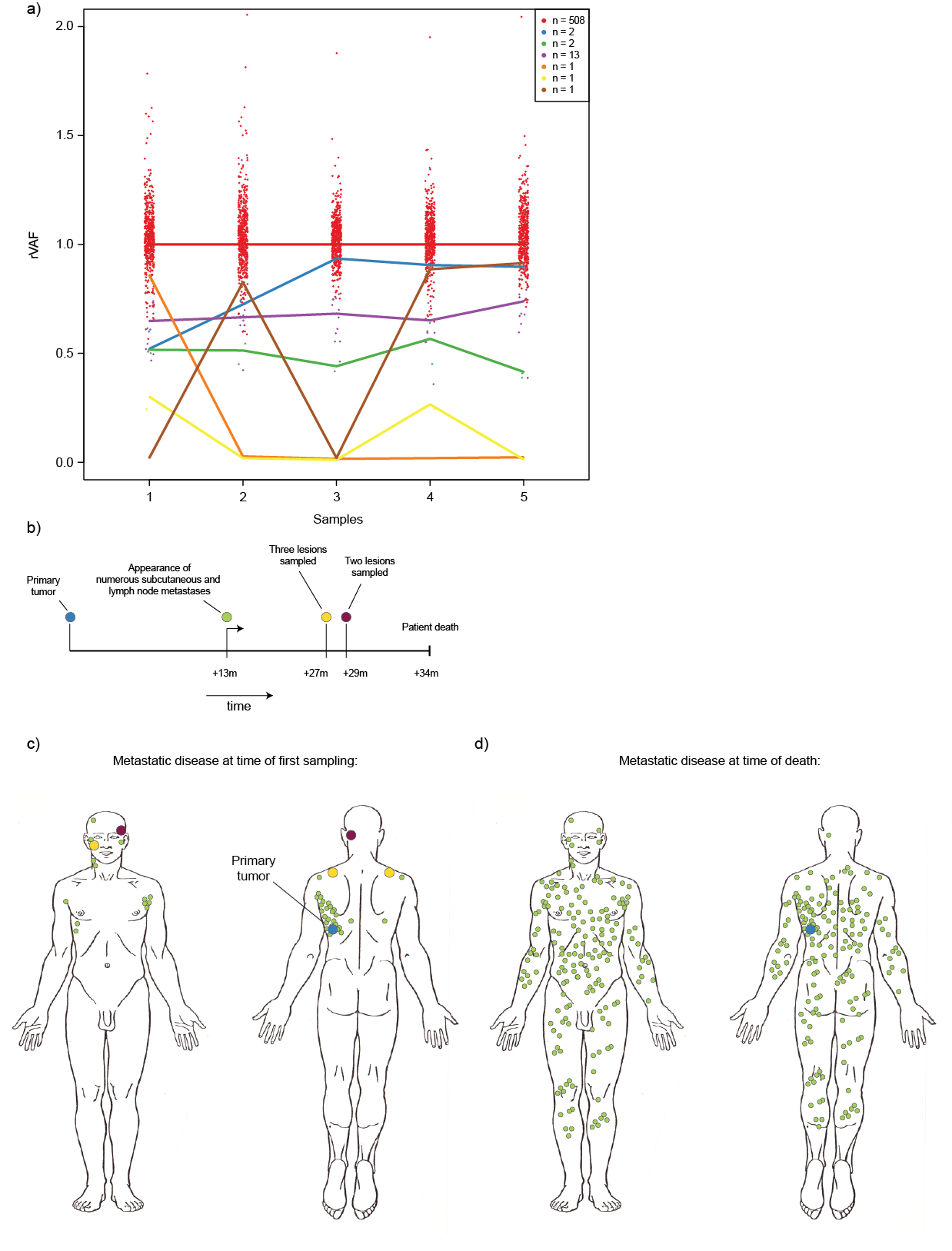


**Supplementary Figure 10** (previous page): Mutations with a recurrent subclonal rVAF could indicate reseeding between lesions. **a**) Cross sample clustering of mutations according to cellular prevalence^1^ as in Supplementary Fig. 9. The number of mutations on which the inference of each cell population is based, are shown in a panel in the top right corner. **b-d**) Patient MM61 had an unusual disease course, characterized by an extensive and rapid spread of cutaneous metastases, first described 13 months following surgical excision of the primary lesion. At the time point of sampling (c) of the first metastatic lesions (sample 1-3) for the current study, regional and distant skin metastases were present on the truncus, neck and upper extremity. Wide-ranging cutaneous progression occurred in the months leading up to the patient’s death, 7 months later (d).


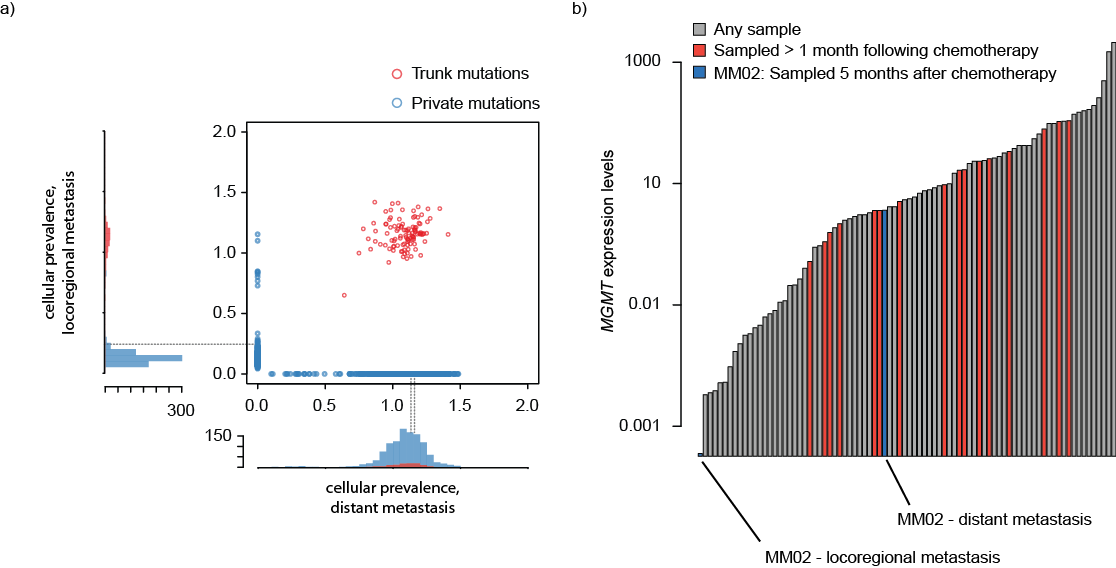


**Supplementary Figure 11**: *MSH6* and *MGMT* deficiency in patient MM02. **a**) Cellular prevalence of mutations in each sample from MM02. The lower and left panels show the frequency distributions of private (blue) and trunk (red) mutations in each sample. These correspond to clonal populations and each major peak of mutations coincides with private *MSH6* mutations, which are indicated with stapled lines. Private mutations in both samples conformed to the dacarbazine signature. **b**) Expression levels of *MGMT* (O-6-methylguanine-DNA methyltransferase) mRNA relative to those of *B2M* (beta-2-microglobulin) were measured for each sample, and compared to the relative *MGMT* expression levels in the melanoma cell line Sk-Mel-28. Samples collected in excess of one month following dacarbazine exposure are shown in red and samples from MM02 in blue. All other samples are shaded gray. Both samples from MM02 displayed a mutational signature consistent with dacarbazine treatment. One sample from MM02 displayed a loss of *MGMT* expression, whereas the other was found to have hypermethylation of the *MGMT* promoter (results not shown). Expression levels and promoter methylation status of *MGMT* for this patient cohort have been published previously ^2^.


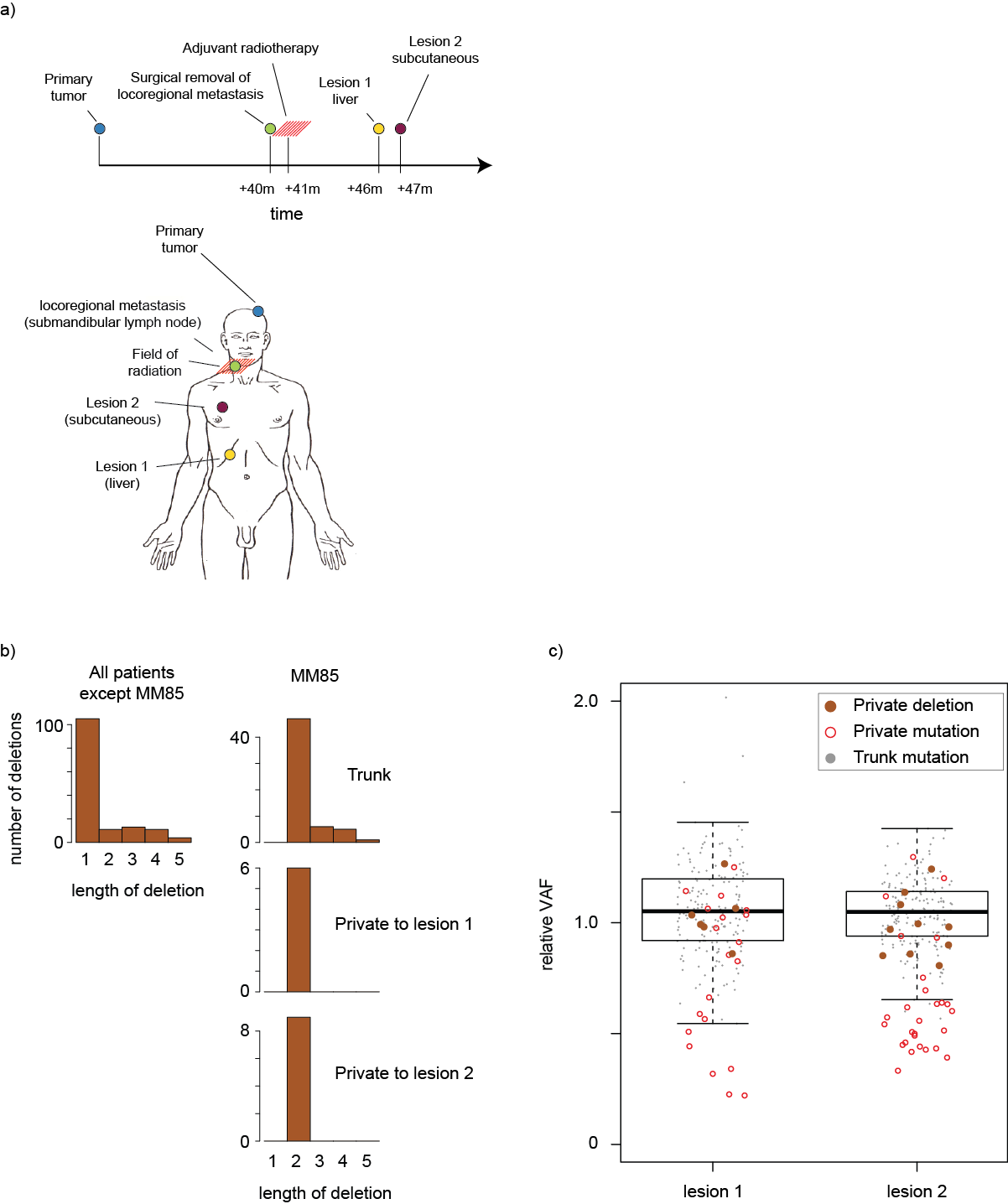


**Supplementary Figure 12**: Mutational pattern of 2-nt deletions in patient MM85. **a**) The upper panel shows the timing of disease progression and radiation treatment (m = months). The lower panel shows the localization of each lesion and the field of radiation. Lesion 1 and lesion 2 were sampled for the current study. **b**) The frequency of deletions according to the length of deletions in all patients except MM85 (upper left), and deletions in MM85; trunk deletions, or deletions private to each lesion (top to bottom, right). **c**) Relative VAF of trunk mutations (gray), private mutations other than deletions (red circles), and private deletions (brown). Boxes are based on the trunk mutations only (as in Supplementary Figure 8).


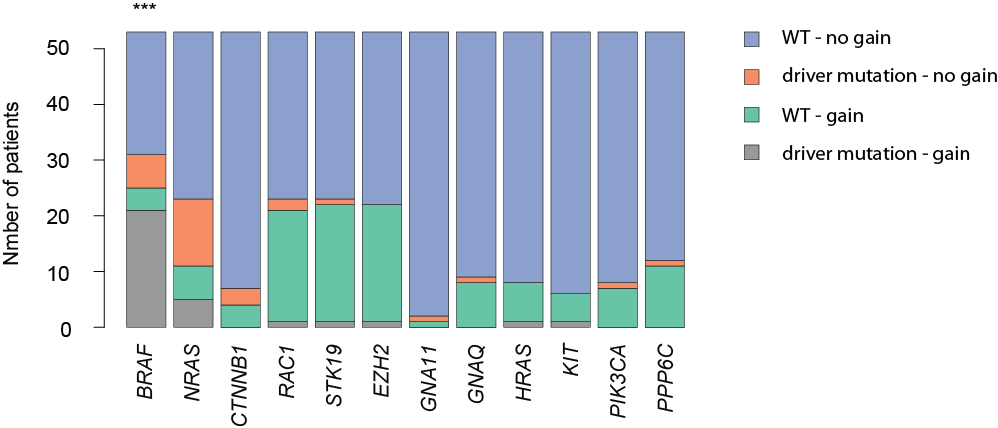


**Supplementary Figure 13**: Frequency of gains of activating driver mutations. Patients are categorized according to whether there is evidence of an increase in copy number of one allele relative to a balanced state of each gene, and whether a driver mutation is identified in each gene. Asterisks indicate a significant difference according to a fisher exact test (p < 0.001).

**Supplementary Figure 14:** Estimation of empirical p-values for random difference in *f* values between segments exceeding the chosen threshold (0.018). (a) Probability density plot of global random differences (x-axis). The gray arrow indicates the *f* value difference corresponding to the global empirical p value 0.05. The black arrow indicates the BAF difference of 0.018 corresponding to the global empirical p value 0.017. (b) Plot of local empirical p values for the cutoff at each specific *f* value. The x-axis shows *f* values ranging from 0.01 to 1, with with an increment of 0.01. For the black line, the y- axis is the local empirical p value corresponding to the difference 0.018 in each specific *f* value. For the red line, the y-axis is the difference of *f* value corresponding to the local empirical p value was 0.05.

**Supplementary Figure 15** The false positive rate (FPR) and false negative rate (FNR) of copy number calling, as functions of tumor purity.

## Supplementary References

1 Roth, A. *et al.* PyClone: statistical inference of clonal population structure in cancer. *Nat Methods* **11**, 396-398, doi:10.1038/nmeth.2883 (2014).

2 Busch, C., Geisler, J., Lillehaug, J. R. & Lonning, P. E. MGMT expression levels predict disease stabilisation, progression-free and overall survival in patients with advanced melanomas treated with DTIC. *Eur J Cancer* **46**, 2127-2133, doi:10.1016/j.ejca.2010.04.023 (2010).
