# Supplementary material for: Patterns of genomic evolution in advanced melanoma
Source: Nat Commun. 2018 Jul 10;9:2665. doi: 10.1038/s41467-018-05063-1 (PMC6039447; doi:10.1038/s41467-018-05063-1)
Supplement: Supplementary file 3 — Description of Additional Supplementary Files [file 41467_2018_5063_MOESM3_ESM.pdf]

Description of Additional Supplementary Files

File Name: Supplementary Data 1
Description: All detected somatic mutations.

File Name: Supplementary Data 2
Description: Detailed presentation of individual patients included in the study including listing of the primary and metastatic deposit locations.

File Name: Supplementary Data 3
Description: Detailed presentation of individual metastatic deposits with previous anti-tumor treatment.

File Name: Supplementary Data 4
Description: Mutations in melanoma driver genes.
